# Infantile Scurvy

**Published:** 1895-08

**Authors:** Irving M. Snow

**Affiliations:** Buffalo. N. Y., Member American Pediatric Society; Physician to Buffalo Fresh Air Mission Hospital.


### INFANTILE SCURVY.

By IRVING M. SNOW, M. D., Buffalo, N. Y.,
Member American Pediatric Society ; Physician to Buffalo Fresh Air Mission Hospital.

In February, 1894, I was called to see a sick baby in the family of a well-to-do German contractor. My patient was the youngest of several children. The mother nursed the baby for one month and afterward fed it on a proprietary dried milk food. Nevertheless the baby had always been delicate and ailing and had never taken its food in a satisfactory way. Still, for a number of months it had no cough or diarrhea nor did it suffer from any acute infectious process. At the age of eleven months the mother noticed that the baby's limbs were very tender and that it cried when she handled or bathed it. From this time on the child grew weaker and more fretful. It was with difficulty induced to take its bottle, slept but little and lay with its legs flexed upon the abdomen. Notwithstanding the child's failing health dentition pursued its normal course, the teeth appeared in regular order during the tenth, eleventh and twelfth months. About February 1st, the baby being over a year old, the family physician found that there was an extensive livid swelling of the gums of the upper jaw. This swelling rapidly increased, the gums bled freely at the slightest touch, the breath grew fetid and a foul-smelling nasal discharge appeared. After this the general condition of the child grew more unfavorable, it refused to swallow and became continually weaker and more apathetic.

As the mouth symptoms resisted all treatment the family physician, thinking that he might have to deal with a cancrum oris, or a necrosis of the alveolar processes, called a surgeon in consultation. The surgeon examined the mouth and refused to interfere, declaring that operative treatment would do no good.

On February 19th I was called in consultation. The child was lying languidly in its mother's arms. On our entrance into the room it set up a piteous wail, evidently anticipating that our object was to hurt her mouth. She was of good length, but emaciated to the last degree, weighing only thirteen and one-half pounds, although thirteen months old. The temperature was 100°; pulse, 120, weak ; the face and skin were of a sallow, muddy pallor; the lips were white; a few purpuric spots, the size of a pin's head, were scattered over the body, arms and legs.

But it was about the mouth that the whole interest of the case centered. On the upper jaw there was an enormous fleshy swelling of the gums. This swelling bulged downward, covering two-thirds of the free surface of the incisor teeth. A similar puffiness was seen about the site of a molar that had fallen out the day before. There was an extensive ecchymosis on the gums on the left side of the lower jaw, while the gums themselves were livid in color and bleeding freely. The mucous membrane of the rest of the buccal cavity was of normal color and free from exudate. No enlargement of the cervical glands was present. On the thorax there was well-marked rachitic beading of the ribs. The legs were thin and flabby, but at this time they were not swollen or tender on manipulation. Just before the consultation the child had vomited blood, which probably came from the diseased gums. A condition of constipation was, perhaps, caused by the semi-starvation of the child. As to the previous treatment the baby was taking a dried milk preparation for food, the medication was iron and gentian, cod liver oil, brandy and chlorate of potash. The family physician, believing that the child was suffering from blood poisoning, had given a most unfavorable prognosis and the parents had lost hope for the baby's recovery.

To review the case—a child reared on a dried milk food was dying of inanition with swollen, bleeding gums. The puny condition of the baby during the first year of life was due to rickets caused by taking a food rich in starch and poor in fats. After ten months of this fare scorbutic mouth symptoms appeared.

I made a diagnosis of infantile scurvy and in spite of the desperate condition of the child told the mother her baby would certainly live. I ordered orange juice, mashed potatoes and a cream mixture to displace the proprietary food. All drugs and antiseptics to the mouth were discontinued. Four days afterward I again saw the child, a great improvement had taken place, the orange juice was greedily swallowed, the baby crying for more. Through some misunderstanding the potato was not given. There was also a noticeable increase in strength, the baby was no longer tender to handle, it sat up and viewed passing events with interest. The mouth was well, the swelling had subsided, no bleeding from the gums had occurred in two days. My diagnosis was proven by the therapeutic test,—recovery after the use of antiscorbutics.

In other ways the baby gained more slowly. The emaciation and digestive disturbance were too grave to disappear at once, yet in a month, with absolutely nothing but dietetic treatment, the child was practically well. Such is the history of the only case of infantile scurvy as yet reported in this city.

Infantile scurvy cannot be regarded as a common malady in this country, as Dr. Northrup, in February, 1894, after an extensive correspondence, was able to collect only 106 American cases.

I have invited attention to a disease whose etiology is understood and whose treatment has been perfected. The cause is probably biological and chemical,—deprivation of fresh food. A cure follows the administration of fresh fruits, vegetables and meat.

In 1883, Drs. Barlow and Cheadle first proved that scurvy occurred among infants in cities as well as among seamen. A baby, even amidst luxurious surroundings, may as easily develop scurvy if it be fed on a dried milk food or condensed milk as a sailor from eating salt pork and hard tack in the polar seas. As a result of a high civilisation the disease has disappeared among sailors, but is now found among the children of the well-to-do. Nearly all cases of infantile scurvy have been discovered in wealthy families, where the parents were able to buy expensive proprietary foods of malted grain and dried milk.

Poor children are given potatoes, an admirable antiscorbutic, and other articles of adult diet at a very early age. It is reasonable to suppose that infantile scurvy is steadily on the increase, as the operating cause, feeding of babies on patent foods, grows more frequent. As I believe that scurvy in children is occasionally overlooked and as a knowledge of its symptoms may be the means of saving life, a review of the various phases of the disease may not be amiss.

Scorbutic babies are generally rachitic; not that rickets is a part of the disease, but simply exists as an associated condition.

At first scurvy was confused with rheumatism on account of the swelling and tenderness of the limbs and joints, hence the condition was long known to the Germans as acute rickets. The German cases were described as swollen, tender limbs in rickety babies. No mouth symptoms were present.

Scurvy has been mistaken for purpura hemorrhagica from the ecchymosis of the skin, or for a stomatitis from the bleeding and sponginess of the gums. Older children occasionally develop scurvy. They are on an adult diet, but show a curious hysterical dislike to fresh fruit and vegetables. No well authenticated case of scurvy in a breast-fed baby is recorded. An infant nursed by a scorbutic mother may develop scurvy.

The symptoms of the disease are found (1) in the mouth and (2) in the limbs. With babies the classical signs of scurvy do not always develop in regular order; thus a baby may have spongy, swollen gums and no pronounced limb symptoms, as in my case, or there may be tense painful swelling of the limbs with no mouth symptoms, or both regions may be simultaneously affected.

As a matter, of fact the sore gums are frequently absent in young infants with no teeth. For this there is a physiological reason. Before the eruption of the teeth the gums have a scanty blood supply and there is relatively little nutrition. Each tooth adds a new lease of blood-vessels to the mucosa of the gums. Almost invariably there is a progressive sallow anemia, caused by recurring hemorrhage, with languor, faintness and other evidences of cardiac weakness. The children affected are often fat and would be called well nourished. The temperature is exceedingly erratic; it may be febrile, normal or subnormal. Vomiting and diarrhea are seldom observed.

The primary scorbutic bone lesion is a hemorrhage between the actively growing periosteum and underlying bone. A thin sheath of blood-clot surrounds the shaft of the bone. A hard, brawny swelling in a thigh or leg is due to an effusion of blood in the deeper layers of muscles or serum into the superficial layers. There is neither local heat nor feeling about the swelling. A loss of power in a limb or a pseudo-paralysis may be the result of the separation of an epiphysis, or caused merely by pain on moving the leg.

Among the most curious features of infantile scurvy is a black eye from a hemorrhage into the loose tissue of the lid, or proptosis bulging of the eyeball from a bloody effusion into the orbital periosteum behind the eye. Extensive purpura, visceral hemorrhages, epistaxis or hematuria have been observed.

Numerous fatal cases of scurvy are recorded, the condition being recognised only when the child was moribund or dead; the post-mortem findings being intramuscular, visceral or subperiosteal hemorrhage.

It is interesting to note that scurvy does not quickly develop in a baby fed in the most irrational manner; the organism seems to live on its fresh tissues for a while. If the disease be untreated, its duration is from two months to four months, and terminates either in death or a slow recovery, as by accident antiscorbutics are given.

Lastly, it is probable that we see and pass unrecognised numerous borderland cases in rickety babies; they are tender to handle and irritable out of all proportion to the signs found. A final diagnosis is made by the therapeutic test, a rapid cure following the use of living food, orange juice, potatoes, meat and fresh milk.

The following case is illustrative:

A child with scurvy was received by Dr. Cheadle into the Great Ormond Street Hospital for Sick Children. The spongy swelling of the gums was so characteristic that Dr. Cheadle determined to have a sketch made. The day was Friday, the artist could not come until Monday. In the meantime the patient was given potato, raw meat, fresh milk. When the artist appeared, three days after he was summoned, the child was so much better, the swelling had so completely subsided that the sketch was abandoned.

476 FRANKLIN STREET.

